# Inflammatory burden index (CRP×NLR) is associated with moderate-to-severe endoscopic activity in hospitalized patients with ulcerative colitis: a single-center retrospective study

**DOI:** 10.1093/crocol/otag073

**Published:** 2026-08-08

**Authors:** Zihan Xu, Shiqing Yuan, Zhen Hu, Jiarun Qian, Lihong Gu, Xiaoyun Wang

**Affiliations:** Wuxi School of Medicine, Jiangnan University, Wuxi, Jiangsu 214000, China; Division of Gastroenterology, Jiangnan University Medical Center (Wuxi No. 2 People’s Hospital), Wuxi, Jiangsu 214000, China; Wuxi School of Medicine, Jiangnan University, Wuxi, Jiangsu 214000, China; Division of Gastroenterology, Jiangnan University Medical Center (Wuxi No. 2 People’s Hospital), Wuxi, Jiangsu 214000, China; Division of Gastroenterology, Jiangnan University Medical Center (Wuxi No. 2 People’s Hospital), Wuxi, Jiangsu 214000, China; Wuxi School of Medicine, Jiangnan University, Wuxi, Jiangsu 214000, China; Division of Gastroenterology, Jiangnan University Medical Center (Wuxi No. 2 People’s Hospital), Wuxi, Jiangsu 214000, China; Division of Gastroenterology, Jiangnan University Medical Center (Wuxi No. 2 People’s Hospital), Wuxi, Jiangsu 214000, China; Division of Gastroenterology, Jiangnan University Medical Center (Wuxi No. 2 People’s Hospital), Wuxi, Jiangsu 214000, China

**Keywords:** inflammatory burden index, ulcerative colitis, Mayo endoscopic subscore, C-reactive protein, neutrophil-to-lymphocyte ratio

## Abstract

**Background:**

In hospitalized patients with ulcerative colitis (UC), timely endoscopy may be constrained. We evaluated whether the inflammatory burden index (IBI; C-reactive protein × neutrophil-to-lymphocyte ratio [CRP×NLR]) is associated with endoscopic activity.

**Methods:**

We retrospectively studied adults hospitalized with UC between 2023 and 2025 who underwent index lower endoscopy during the same admission, with blood sampling within 24 hours of admission and endoscopy within 72 hours of admission. Endoscopic activity was graded by Mayo Endoscopic Subscore (MES) and dichotomized as MES 0-1 versus ≥2. Associations with MES ≥2 were examined using logistic regression with adjustment for age, sex, and body mass index, with IBI modeled as log(IBI + 1). Discrimination was assessed by receiver operating characteristic analysis with same-sample DeLong testing. Sensitivity analyses were restricted to endoscopy within 48 hours after blood sampling.

**Results:**

Among 107 patients, 83 (77.6%) had MES ≥2. IBI correlated with MES (ρ  =  0.58; *P* < .001) and remained independently associated with MES ≥2 (adjusted OR, 5.28; 95% CI, 2.12-13.14; *P* < .001). Discrimination was reasonable (AUC, 0.83; 95% CI, 0.76-0.90). CRP alone performed similarly (AUC, 0.80; 95% CI, 0.71-0.88; *P* = .227 vs IBI), as did a CRP+NLR model (AUC, 0.84; 95% CI, 0.77-0.91; *P* = .40 vs IBI). Findings were directionally consistent in the ≤48-hour sensitivity analysis. The Youden-derived IBI cutoff was 3.65 (sensitivity, 72.3%; specificity, 91.7%) and should be considered exploratory.

**Conclusions:**

In this cohort, IBI was associated with moderate-to-severe endoscopic activity. Performance was comparable to CRP alone, and findings should be interpreted as exploratory rather than as evidence for a standalone clinical decision tool.

## Graphical abstract

**Graphical Abstract:**
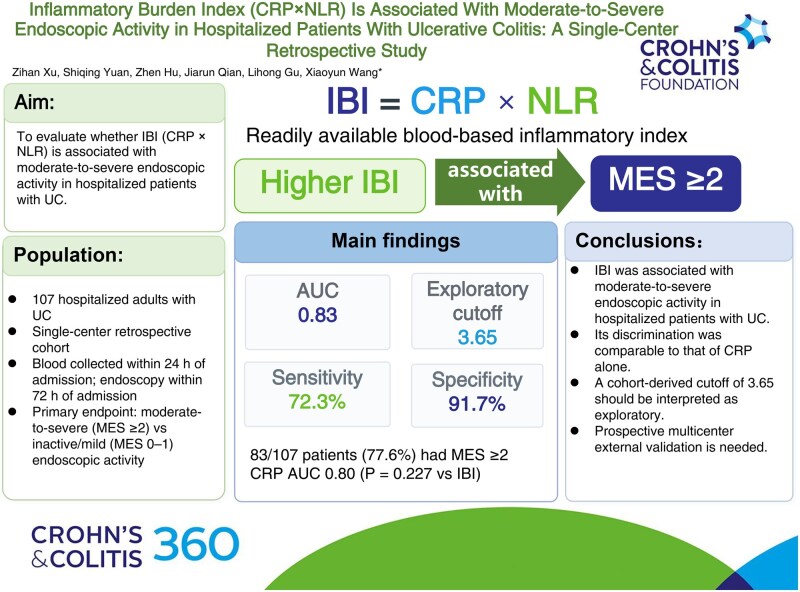

## Introduction

Ulcerative colitis (UC) is a chronic relapsing inflammatory bowel disease in which endoscopic healing is a key therapeutic target under treat-to-target strategies, including STRIDE-II.^1^ Colonoscopy remains the reference standard for grading disease activity and assessing treatment response.^2^ In hospitalized patients with acute exacerbations, however, repeated endoscopic reassessment is often difficult because of clinical instability, bowel preparation concerns, and limited endoscopy availability.^3^ Clinicians therefore often rely on surrogate markers for short-term assessment of inflammatory burden, highlighting the need for a simple and readily available marker of mucosal activity in the inpatient setting.^4^

Conventional blood biomarkers, including C-reactive protein (CRP), erythrocyte sedimentation rate (ESR), and the neutrophil-to-lymphocyte ratio (NLR), are widely used but typically show only moderate correlations with endoscopic activity (Spearman’s ρ approximately 0.40-0.55 in previous reports), which may limit their ability to reflect residual mucosal inflammation accurately.^5^ In contrast, fecal calprotectin (FC) is a well-validated noninvasive marker of UC activity that correlates closely with endoscopic severity.^6^^,^^7^ Schoepfer et al. reported stronger correlations of FC with endoscopic activity than those observed for conventional blood markers in UC,^5^ and Theede et al. showed that FC correlates with both endoscopic and histologic inflammation and can identify mucosal healing.^8^ However, in inpatient practice, FC testing may be constrained by difficulties in timely stool collection, delays in laboratory turnaround, and lack of routine availability in many hospitals.^9^^,^^10^ Consequently, readily available biomarkers still have limitations for rapid noninvasive assessment of mucosal activity in hospitalized patients with UC.

The inflammatory burden index (IBI), defined as CRP × NLR, integrates the acute-phase response and leukocyte redistribution into a single composite measure of systemic inflammation.^11^ IBI has shown prognostic value in several inflammatory conditions and has been explored as a composite inflammatory marker in settings such as sepsis^12^ and coronary artery disease.^13^ However, its association with endoscopic activity in UC, particularly among hospitalized patients, has not been well characterized. Moreover, it remains unclear whether IBI provides information that is complementary to conventional blood markers used in UC, including CRP, ESR, NLR, platelet-to-lymphocyte ratio (PLR), and lymphocyte-to-monocyte ratio (LMR).^10^^,^^14^^,^^15^

Therefore, we conducted a single-center retrospective study of hospitalized patients with UC who underwent index lower endoscopy during the same admission, with blood samples collected within 24 hours of hospital admission and index lower endoscopy performed within 72 hours of hospital admission. We aimed (i) to examine the association between IBI and Mayo Endoscopic Subscore (MES)-graded endoscopic activity; (ii) to compare the discriminatory performance of IBI with conventional blood biomarkers (CRP, ESR, NLR, and PLR) for identifying moderate-to-severe endoscopic activity (MES ≥2); and (iii) to assess whether IBI remained independently associated with MES ≥2 after adjustment for age, sex, and body mass index.

## Materials and methods

### Study design and patients

This single-center retrospective observational study included consecutive adult (≥18 years) patients hospitalized with UC who underwent index lower endoscopy (full colonoscopy or flexible sigmoidoscopy) during the same admission at Jiangnan University Medical Center (Wuxi No. 2 People’s Hospital) between January 1, 2023, and December 1, 2025. Data abstraction was completed on December 1, 2025. To avoid duplicate observations, only the first eligible admission per patient was included.

UC was confirmed using standard clinical, endoscopic, and histological criteria in accordance with the ECCO consensus guidelines.^16^ Eligible patients had (i) index lower endoscopy with documentation sufficient to assign a Mayo Endoscopic Subscore (MES)^17^ and (ii) blood samples collected within 24 hours of hospital admission, including complete blood count with differential and CRP, with index lower endoscopy performed within 72 hours of hospital admission during the same admission. For cohort eligibility, both blood sampling and index lower endoscopy were anchored to hospital admission. Separately, the observed blood-draw-to-endoscopy interval was examined as a secondary timing variable and used for sensitivity analyses. This temporal pairing was prespecified so that both blood sampling and endoscopic assessment were anchored to the time of hospital admission and were intended to reflect the same hospitalization-related inflammatory episode. Because blood sampling was obtained early after hospitalization and endoscopy was restricted to an early prespecified window during the same admission, the paired measurements were considered to reflect the same admission-related inflammatory state. During this interval, treatment exposure primarily reflected baseline/admission treatment background or very early inpatient therapy rather than prolonged interval treatment. Over such a short timeframe, treatment-related changes may be more readily captured in circulating inflammatory indices than in endoscopic mucosal severity, which generally evolves more slowly. Nevertheless, the precise sequencing of treatment changes between blood draw and endoscopy could not be reliably determined from the retrospective source records and was not analyzed separately. ESR was recorded when available.

Patients were excluded if the index lower endoscopy was incomplete or insufficiently documented for MES grading; if CRP or differential leukocyte counts from blood samples collected within 24 hours of hospital admission were unavailable; or if major comorbid conditions likely to substantially affect systemic inflammatory markers were present (e.g., severe trauma, decompensated cirrhosis, end-stage renal disease/uremia, pregnancy, active malignancy, or severe extraintestinal infection such as pneumonia or sepsis not attributable to UC). Patients with Crohn’s disease, inflammatory bowel disease unclassified, ischemic or infectious colitis, other specific colitides, colorectal cancer, or chronic systemic autoimmune diseases (e.g., psoriasis, Behçet’s disease, rheumatoid arthritis, systemic vasculitis) were also excluded.

Clinical severity at admission was unavailable for 9 patients (8 in the MES 0-1 group and 1 in the MES ≥2 group) because of incomplete documentation; these patients were excluded only from analyses involving clinical severity and were retained in all other analyses.

### Endoscopic assessment

Index endoscopic assessments included both full colonoscopy and flexible sigmoidoscopy, according to inpatient practice and procedural feasibility.^18^ Endoscopic activity was graded retrospectively using the Mayo Endoscopic Subscore (MES, 0-3) according to standard definitions, based on the original report and available images; the assigned score reflected the most severe mucosal finding documented within the examined segment(s).^17^ Grading was performed by an experienced endoscopist blinded to laboratory data, and only examinations with documentation sufficient for MES assignment were included.

Intra-rater reliability was assessed by repeat scoring of a random subset (*n* = 21) at least 4 weeks later using a computer-generated sequence, blinded to initial scores.^19^ Agreement was excellent (quadratic-weighted Cohen’s κ  =  0.85).

### Clinical and laboratory data

Demographic and baseline characteristics were extracted from electronic records, including age, sex, body mass index (BMI), and disease extent according to the Montreal classification (E1-E3).^20^ Clinical disease severity at admission was categorized as mild, moderate, or severe when explicitly documented in the medical record according to standardized criteria (e.g., Truelove and Witts criteria^21^ and/or Mayo clinical subscore^17^^,^^22^) based on symptoms, physical findings, and routine laboratory parameters. When such criteria were not documented, clinical severity was treated as missing and excluded only from analyses involving clinical severity.

Laboratory data were obtained from blood samples collected within 24 hours of hospital admission. Index lower endoscopy was performed within 72 hours of hospital admission. Parameters included white blood cell count, neutrophil/lymphocyte/monocyte counts (×10^9^/L), platelet count (×10^9^/L), and CRP (mg/L). CRP was measured as part of routine clinical laboratory testing, and the local upper limit of normal (ULN) was 10 mg/L. ESR (mm/h) was analyzed in the subset with available measurements. FC was not routinely obtained and was not analyzed. BMI was calculated from admission measurements when available; patients with missing BMI were excluded only from BMI-adjusted models. Exact continuous timestamps for blood draw and endoscopy were not consistently available across the full cohort; however, the blood-draw-to-endoscopy interval could be categorized into prespecified timing windows, and a sensitivity analysis was performed in patients who underwent endoscopy within 48 hours after blood sampling. Thus, admission-based timing defined cohort eligibility, whereas the blood-draw-to-endoscopy interval was analyzed separately to assess robustness to narrower observed sampling windows. Because blood sampling occurred early after admission and endoscopy was performed within an early prespecified window during the same hospitalization, these paired assessments were intended to capture the same hospitalization-related inflammatory episode. Over this timeframe, treatment-related changes may be more readily reflected in circulating inflammatory indices than in endoscopic mucosal appearance, which generally evolves more slowly. Exact treatment sequencing between blood draw and endoscopy, including corticosteroids, rescue therapies, antibiotics, and biologic/JAK exposure, was not reliably recoverable from the retrospective source records and was therefore not analyzed separately.

### Inflammatory indices

Derived indices were calculated as follows: neutrophil-to-lymphocyte ratio (NLR) = neutrophils/lymphocytes; platelet-to-lymphocyte ratio (PLR) = platelets/lymphocytes; lymphocyte-to-monocyte ratio (LMR) = lymphocytes/monocytes; and inflammatory burden index (IBI) = CRP × NLR.

### Outcome definition

The primary outcome was moderate-to-severe endoscopic activity, defined as MES ≥2 at index lower endoscopy. A sensitivity analysis additionally examined severe endoscopic activity, defined as MES 3. Secondary outcomes were (i) associations between blood markers (CRP, ESR, NLR, PLR, LMR, and IBI) and MES treated as an ordinal variable, and (ii) discrimination of MES ≥2 versus MES 0-1 by IBI and conventional blood markers. For ROC comparisons, we focused on commonly used inflammatory markers (CRP, ESR, NLR, and PLR) to reflect routine clinical practice; ESR analyses were restricted to patients with available ESR measurements.

### Statistical analysis

Normality was assessed using histograms/Q-Q plots and the Shapiro-Wilk test. Skewed continuous variables are presented as median (IQR) and compared using the Mann-Whitney *U* test. Categorical variables are presented as *n* (%) and compared using χ^2^ or Fisher’s exact tests, as appropriate.

Spearman’s correlation (ρ) quantified associations between MES (0-3) and each marker. For Figure 2, 95% confidence intervals for Spearman’s ρ were estimated by bootstrap resampling. For regression analyses, skewed markers were log-transformed as log(x + 1). Logistic regression evaluated associations with MES ≥2: univariable models were fitted first, followed by a multivariable model including log(IBI + 1) adjusted for age, sex, and BMI. Additional adjustment for comorbidities was not performed because major comorbid conditions expected to materially affect inflammatory markers had already been excluded by design, and the retrospective sample size was limited. An exploratory multivariable model including log(CRP + 1) and log(NLR + 1), adjusted for age, sex, and BMI, was also constructed to provide a benchmark for discrimination analyses comparing IBI with a two-variable CRP+NLR model. Sensitivity analyses were performed using MES 3 as the endpoint and, separately, in patients who underwent endoscopy within 48 hours after blood sampling to assess robustness to a narrower sampling window. Internal assessment of standalone IBI was additionally performed using bootstrap resampling to evaluate discrimination and the within-cohort stability of the cohort-derived cutoff (3.65).

Discrimination for MES ≥2 was assessed using ROC curves and AUCs with 95% CIs; ROC curves for IBI and comparator markers were compared using same-sample DeLong testing for correlated AUCs. Optimal cutoffs were derived using Youden’s index, with sensitivity, specificity, positive and negative likelihood ratios, and corresponding 95% confidence intervals reported. Internal validation and calibration of the multivariable model were assessed by bootstrap resampling (section “Internal validation and model calibration”). Decision-curve analysis was performed for standalone IBI, CRP alone, and the multivariable model (section “Decision curve analysis”). Analyses were performed using complete-case data for each model or comparison, and no imputation was performed. Two-sided *P* < .05 was considered statistically significant. Analyses were performed in SPSS (version 26.0) and R (version 4.3) using standard packages (pROC, boot, rms, dcurves).

### Internal validation and model calibration

Bootstrap internal validation (500 resamples) was used to estimate optimism in discrimination for the multivariable model (log(IBI + 1), age, sex, BMI). Optimism was defined as the mean difference between the apparent AUC in the bootstrap sample and the test AUC when the model was applied to the original dataset; an optimism-corrected AUC was computed accordingly. Calibration was summarized by the calibration intercept and slope and visualized with a smoothed calibration curve. For standalone IBI, bootstrap resampling (5000 resamples) was used to assess discrimination and the stability of the Youden-optimal cutoff on the original IBI scale.

### Decision curve analysis

Decision-curve analysis quantified net benefit across threshold probabilities for identifying MES ≥2. Net benefit was evaluated for standalone IBI, CRP alone, and the multivariable model including log(IBI + 1), age, sex, and BMI, each compared with “treat-all” and “treat-none” strategies.^23^ Threshold probabilities from 0 to 0.80 were examined descriptively, with particular attention to mid-to-high threshold ranges.

### Ethics

The protocol was approved by the Ethics Committee/Institutional Review Board of Jiangnan University Medical Center (Wuxi No. 2 People’s Hospital) (Approval No. WXEY-2025-232). Informed consent was waived due to the retrospective use of de-identified data. The study was conducted in accordance with the Declaration of Helsinki.

## Results

### Study population and baseline characteristics

**Figure 1:**
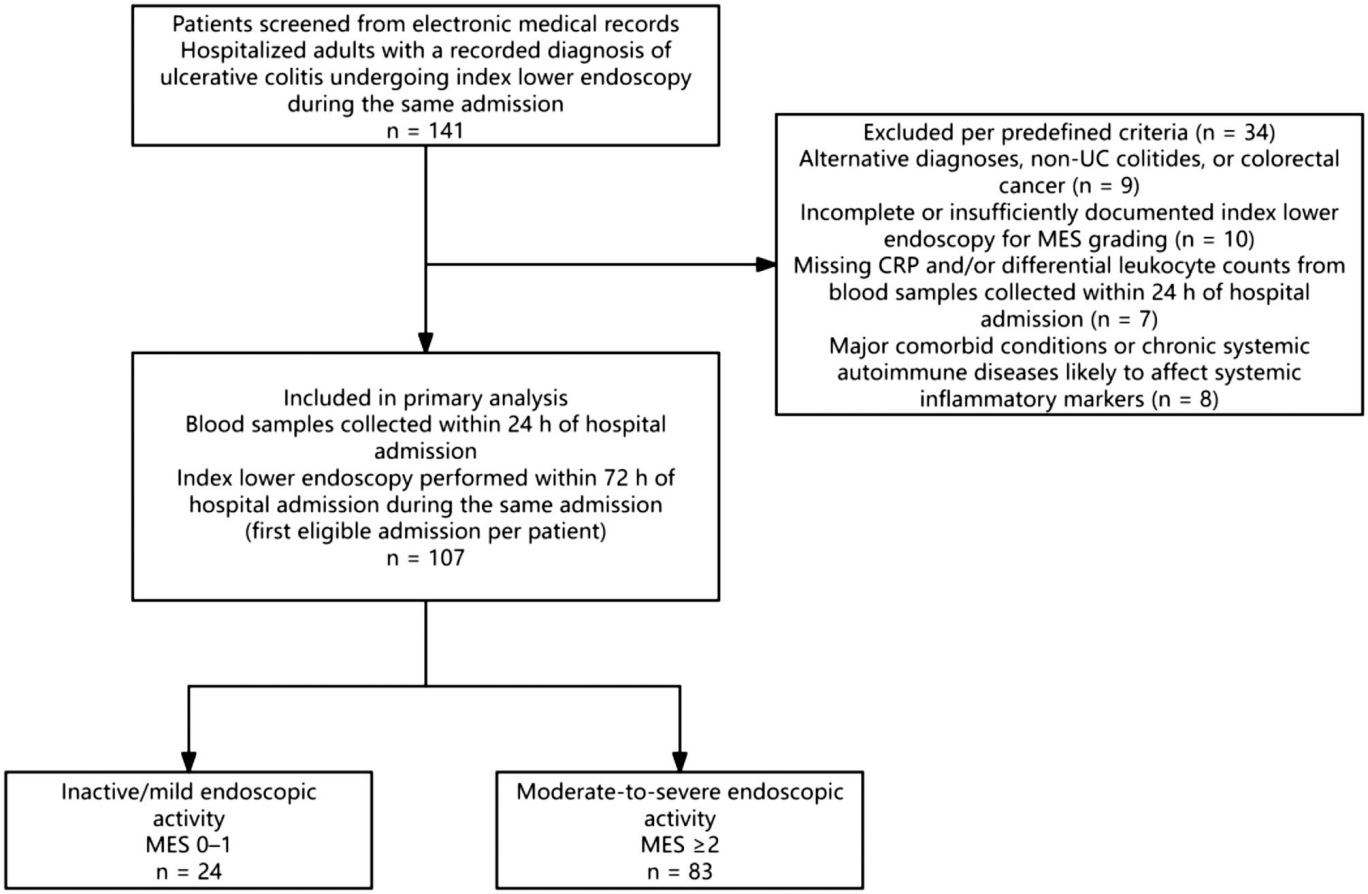
Flow diagram of patient selection and study cohort. Consecutive hospitalized adults with ulcerative colitis undergoing index lower endoscopy during the same admission (January 1, 2023 to December 1, 2025) were screened. After application of predefined exclusion criteria, eligible patients with sufficient endoscopic documentation for Mayo Endoscopic Subscore (MES) assignment, blood samples collected within 24 hours of hospital admission, and index lower endoscopy performed within 72 hours of hospital admission were included (first eligible admission per patient).

During the study period, 141 consecutive hospitalized adults with UC who underwent index lower endoscopy were screened for eligibility. After applying the predefined eligibility criteria, 107 patients were included in the final analysis (Figure 1). A total of 24 patients (22.4%) had inactive/mild endoscopic activity (MES 0-1), whereas 83 (77.6%) had moderate-to-severe endoscopic activity (MES ≥2). The distribution of Mayo Endoscopic Subscores was MES 0 in 7 patients, MES 1 in 17 patients, MES 2 in 35 patients, and MES 3 in 48 patients (Table S1). Blood samples used for analysis were collected within 24 hours of hospital admission, and index endoscopy was performed within 72 hours of hospital admission. Separately, in the structured dataset, the observed blood-draw-to-endoscopy interval was available as categorical timing windows rather than exact timestamps. The blood-to-endoscopy interval was within 24 hours in 19 patients (17.8%), >24 to 48 hours in 45 (42.1%), and >48 to 72 hours in 43 (40.2%) (Table S2). Overall, 64 patients (59.8%) underwent endoscopy within 48 hours after blood sampling.

Demographic characteristics and disease extent were broadly comparable between groups (Table 1). The median age of the cohort was 55.0 years (interquartile range [IQR] 46.2-64.0), and 60.7% of patients were male. No significant between-group differences were observed in age (55.0 vs 55.0 years; *P* = .464), sex distribution (54.2% vs 62.7% male; *P* = .608), or body mass index (BMI; 22.6 vs 22.5 kg/m^2^; *P* = .788). Disease extent by Montreal classification was also similar (*P* = .926), with extensive colitis (E3) being the most common phenotype (58.9%). Baseline/admission treatment background was generally similar between groups for 5-ASA use (*P* = .870) and biologic/JAK inhibitor use (*P* = .908), whereas corticosteroid use at admission was more frequent in patients with MES ≥2 than in those with MES 0-1 (31.3% vs 0.0%, *P* < .001).

**Table 1:** Baseline and admission characteristics of hospitalized patients with ulcerative colitis according to Mayo Endoscopic Subscore.

Variable	Total (*n* = 107)	Inactive/mild (MES 0-1, *n* = 24)	Moderate-to-severe (MES ≥2, *n* = 83)	Z/χ²	*P* value
**Age, years**	55.0 (46.2-64.0)	55.0 (46.2-59.0)	55.0 (46.2-65.8)	−0.74	0.464
**Male, *n* (%)**	65 (60.7%)	13 (54.2%)	52 (62.7%)	0.26	0.608
**BMI, kg/m²**	22.5 (19.7-24.2)	22.6 (19.9-24.5)	22.5 (19.7-24.0)	0.27	0.788
**White blood cell count, ×10⁹/L**	5.8 (4.8-8.0)	5.3 (4.2-5.8)	6.4 (5.0-8.3)	−3.12	0.002
**Monocyte count, ×10⁹/L**	0.4 (0.3-0.5)	0.3 (0.2-0.4)	0.4 (0.3-0.6)	−3.05	0.002
**Disease extent (Montreal), *n* (%)**	107	24	83	0.15	0.926
** E1 (proctitis)**	21 (19.6%)	4 (16.7%)	17 (20.5%)		
** E2 (left-sided colitis)**	21 (19.6%)	5 (20.8%)	16 (19.3%)		
** E3 (extensive colitis)**	63 (58.9%)	13 (54.2%)	50 (60.2%)		
** Not recorded**	2 (1.9%)	2 (8.3%)	0 (0.0%)		
**Neutrophil count, ×10⁹/L**	3.7 (2.8-5.3)	2.8 (2.4-3.6)	4.1 (3.1-6.2)	−3.81	<0.001
**Platelet count, ×10⁹/L**	236.0 (196.5-296.0)	220.0 (178.8-264.2)	243.0 (206.0-299.5)	−1.46	0.146
**Lymphocyte count, ×10⁹/L**	1.3 (1.1-1.8)	1.4 (1.2-1.9)	1.2 (1.0-1.8)	1.56	0.120
**NLR**	2.7 (1.8-4.5)	1.9 (1.7-2.5)	3.2 (2.1-5.1)	−3.67	<0.001
**PLR**	161.1 (120.2-241.3)	136.3 (118.0-168.8)	176.6 (125.2-262.8)	−2.33	0.020
**LMR**	3.8 (2.4-4.6)	4.7 (4.1-5.5)	3.4 (2.2-4.4)	3.92	<0.001
**CRP, mg/L**	1.5 (0.5-10.5)	0.5 (0.5-0.9)	2.6 (0.9-18.7)	−4.50	<0.001
**ESR, mm/h (*n* = 101)**	13.0 (7.0-27.0)	7.0 (5.0-12.8)	18.0 (9.0-32.0)	−3.83	<0.001
**IBI**	5.2 (1.3-45.5)	1.0 (0.8-2.3)	10.5 (2.5-64.9)	−4.95	<0.001
**Baseline/admission treatment background, *n* (%)**	107	24	83		
**5-ASA use at admission, *n* (%)**	75 (70.1%)	16 (66.7%)	59 (71.1%)	0.03	0.870
**Any corticosteroid use at admission, *n* (%)**	26 (24.3%)	0 (0.0%)	26 (31.3%)	—	<0.001
**Biologic/JAK inhibitor use at admission, *n* (%)**	28 (26.2%)	7 (29.2%)	21 (25.3%)	0.01	0.908
**Clinical severity at admission, *n* (%)**	107	24	83	27.88	<0.001
** Mild**	24 (22.4%)	12 (50.0%)	12 (14.5%)		
** Moderate**	38 (35.5%)	4 (16.7%)	34 (41.0%)		
** Severe**	36 (33.6%)	0 (0.0%)	36 (43.4%)		
** Not documented (missing)**	9 (8.4%)	8 (33.3%)	1 (1.2%)		

Abbreviations: BMI, body mass index; CRP, C-reactive protein; ESR, erythrocyte sedimentation rate; IBI, inflammatory burden index; LMR, lymphocyte-to-monocyte ratio; MES, Mayo Endoscopic Subscore; NLR, neutrophil-to-lymphocyte ratio; PLR, platelet-to-lymphocyte ratio.

Continuous variables are presented as median (interquartile range) and compared using the Mann-Whitney *U* test. Categorical variables are presented as *n* (%) and compared using the χ^2^ test or Fisher’s exact test, as appropriate.

ESR was available in 101 patients; analyses involving ESR were restricted to those with measured values. Clinical severity at admission was documented in 98 patients; χ^2^ testing for clinical severity was performed among patients with documented severity (mild/moderate/severe) only. Patients with missing clinical severity are shown descriptively but were excluded from the test. The overall distribution of Mayo Endoscopic Subscores was as follows: MES 0, *n* = 7; MES 1, *n* = 17; MES 2, *n* = 35; and MES 3, *n* = 48.

Medication variables reflect baseline/admission treatment background rather than the exact sequencing of treatment changes between blood draw and endoscopy; categories are not mutually exclusive.

Clinical severity at admission differed significantly between groups (*P* < .001), with severe clinical presentations observed predominantly in patients with MES ≥2. Because clinical severity was not documented for 9 patients (8 in the MES 0-1 group and 1 in the MES ≥2 group), analyses involving clinical severity were restricted to patients with documented severity (Table 1).

Laboratory inflammatory indices differed according to endoscopic severity. White blood cell count, neutrophil count, and monocyte count were higher in patients with MES ≥2 than in those with MES 0-1 (WBC: 6.4 vs 5.3 × 10^9^/L, *P* = .002; neutrophils: 4.1 vs 2.8 × 10^9^/L, *P* < .001; monocytes: 0.4 vs 0.3 × 10^9^/L, *P* = .002). Platelet counts were numerically higher (243.0 vs 220.0 × 10^9^/L, *P* = .146), whereas lymphocyte counts were slightly lower (1.2 vs 1.4 × 10^9^/L, *P* = .120).

Derived ratios also differed between groups. Median NLR increased from 1.9 (IQR 1.7-2.5) in the inactive/mild group to 3.2 (2.1-5.1) in the moderate-to-severe group (*P* < .001). PLR was higher (176.6 vs 136.3, *P* = .020), whereas LMR was lower (3.4 vs 4.7, *P* < .001) in patients with MES ≥2.

Conventional inflammatory markers and the IBI also differed substantially by endoscopic severity. Median CRP increased from 0.5 mg/L (IQR 0.5-0.9) in the inactive/mild group to 2.6 mg/L (0.9-18.7) in the moderate-to-severe group (*P* < .001). ESR, measured in a subset of 101 patients, was also higher in the moderate-to-severe group (18.0 vs 7.0 mm/h; *P* < .001). IBI also differed between groups, with median values of 1.0 (0.8-2.3) in the inactive/mild group and 10.5 (2.5-64.9) in the moderate-to-severe group (*P* < .001).

### Correlation between blood markers and endoscopic activity

**Figure 2:**
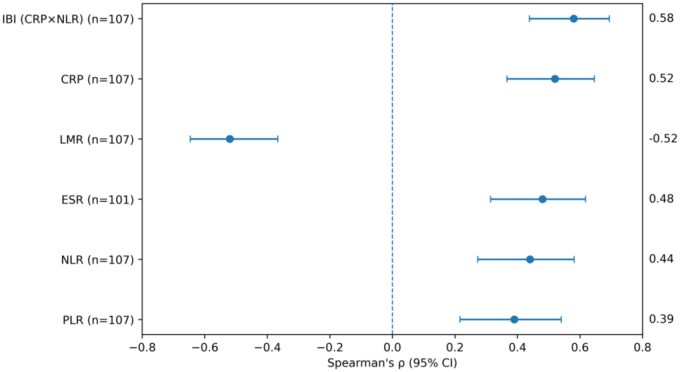
Forest plot of Spearman correlations between blood inflammatory markers and Mayo Endoscopic Subscore (MES) in hospitalized patients with ulcerative colitis. Correlations were assessed using Spearman’s rank correlation, with MES treated as an ordinal variable (0-3). Each horizontal line represents the 95% confidence interval (CI) for Spearman’s ρ, estimated by bootstrap resampling, and the dot indicates the point estimate. All correlations were statistically significant (all *P* < .001). Abbreviations: IBI, inflammatory burden index (CRP × NLR); CRP, C-reactive protein; ESR, erythrocyte sedimentation rate; NLR, neutrophil-to-lymphocyte ratio; PLR, platelet-to-lymphocyte ratio; LMR, lymphocyte-to-monocyte ratio; MES, Mayo Endoscopic Subscore. ESR data were available for 101 patients; other markers were available for 107 patients.

Correlations between endoscopic severity and systemic inflammatory markers are shown in Table 2 and Figure 2. When MES was treated as an ordinal variable (0-3), all evaluated markers were significantly correlated with endoscopic activity (all *P* < .001). Among conventional markers, CRP demonstrated a moderate positive correlation with MES (Spearman ρ = 0.52, *P* < .001; *n* = 107), and ESR, measured in 101 patients, showed a similar positive association (ρ = 0.48, *P* < .001). NLR and PLR were also positively correlated with MES (ρ = 0.44 and 0.39, respectively; both *P* < .001; *n* = 107). In contrast, LMR showed a significant inverse correlation with MES (ρ=−0.52, *P* < .001; *n* = 107).

**Table 2:** Correlation between blood markers and Mayo Endoscopic Subscore.

Marker	Spearman ρ	*P* value	*n*
**Inflammatory burden index (IBI)**	0.58	<0.001	107
**C-reactive protein (CRP), mg/L**	0.52	<0.001	107
**Lymphocyte-to-monocyte ratio (LMR)**	−0.52	<0.001	107
**Erythrocyte sedimentation rate (ESR), mm/h**	0.48	<0.001	101
**Neutrophil-to-lymphocyte ratio (NLR)**	0.44	<0.001	107
**Platelet-to-lymphocyte ratio (PLR)**	0.39	<0.001	107

Correlations were calculated using Spearman’s rank test, with the Mayo Endoscopic Subscore (MES) treated as an ordinal variable (0-3). Abbreviations: IBI, inflammatory burden index; CRP, C-reactive protein; LMR, lymphocyte-to-monocyte ratio; ESR, erythrocyte sedimentation rate; NLR, neutrophil-to-lymphocyte ratio; PLR, platelet-to-lymphocyte ratio. IBI = CRP × NLR; NLR = neutrophil count/lymphocyte count; PLR = platelet count/lymphocyte count; LMR = lymphocyte count/monocyte count. ESR data were available for 101 patients; all other markers were available for 107 patients.

The IBI (CRP × NLR) showed the strongest positive correlation with MES (ρ = 0.58, *P* < .001; *n* = 107) among the blood markers examined.

### Association between IBI and moderate-to-severe endoscopic activity

The association between IBI and the primary binary outcome (moderate-to-severe endoscopic activity; MES ≥2 vs MES 0-1) was evaluated using logistic regression. In univariable analysis, higher IBI was significantly associated with MES ≥2. When expressed as log(IBI + 1), each 1-unit increase was associated with 4.84-fold higher odds of moderate-to-severe endoscopic activity (OR 4.84, 95% CI, 2.08-11.25; *P* < .001) (Table 3). This association remained significant after adjustment for age, sex, and BMI (*n* = 106 with complete data), with log(IBI + 1) independently associated with MES ≥2 (adjusted OR 5.28, 95% CI, 2.12-13.14; *P* < .001), whereas age (OR 1.00 per year, 95% CI, 0.96-1.04; *P* = .986), male sex (OR 1.44, 95% CI, 0.46-4.49; *P* = .531), and BMI (OR 1.08 per kg/m^2^, 95% CI, 0.88-1.32; *P* = .455) were not significantly associated with MES ≥2 (Table 3).

**Table 3:** Logistic regression models for moderate-to-severe endoscopic activity (MES ≥2).

Variable	Model 1 OR (95% CI)	*P*-value	Model 2 OR (95% CI)	*P*-value	Model 3 OR (95% CI)	*P*-value
**log(IBI + 1), per 1-unit increase**	4.84 (2.08-11.25)	<0.001	5.28 (2.12-13.14)	<0.001	–	–
**log(CRP + 1), per 1-unit increase**	–	–	–	–	6.22 (1.79-21.64)	0.004
**log(NLR + 1), per 1-unit increase**	–	–	–	–	8.65 (1.58-47.47)	0.013
**Age, per year**	–	–	1.00 (0.96-1.04)	0.986	1.00 (0.96-1.04)	0.995
**Male sex (vs female)**	–	–	1.44 (0.46-4.49)	0.531	1.51 (0.48-4.76)	0.482
**BMI, per kg/m²**	–	–	1.08 (0.88-1.32)	0.455	1.10 (0.89-1.36)	0.360

Model 1: univariable model including log(IBI + 1) only.

Model 2: model including log(IBI + 1), adjusted for age, sex, and BMI.

Model 3: model including log(CRP + 1) and log(NLR + 1), adjusted for age, sex, and BMI.

Model 1 was fitted in 107 patients; Models 2 and 3 were fitted in 106 patients with complete data on age, sex, and BMI.

Outcome: moderate-to-severe endoscopic activity (MES ≥2).

Abbreviations: OR, odds ratio; CI, confidence interval; IBI, inflammatory burden index; CRP, C-reactive protein; NLR, neutrophil-to-lymphocyte ratio; BMI, body mass index.

To provide an interpretable benchmark, we fitted a parsimonious model including log(CRP + 1) and log(NLR + 1), adjusted for age, sex, and BMI (*n* = 106), in which both components were independently associated with MES ≥2 (log(CRP + 1): OR 6.22, 95% CI, 1.79-21.64; *P* = .004; log(NLR + 1): OR 8.65, 95% CI, 1.58-47.47; *P* = .013) (Table 3). These findings supported subsequent discrimination analyses comparing IBI with a two-variable CRP+NLR model.

### Diagnostic performance of IBI for moderate-to-severe endoscopic activity

**Figure 3:**
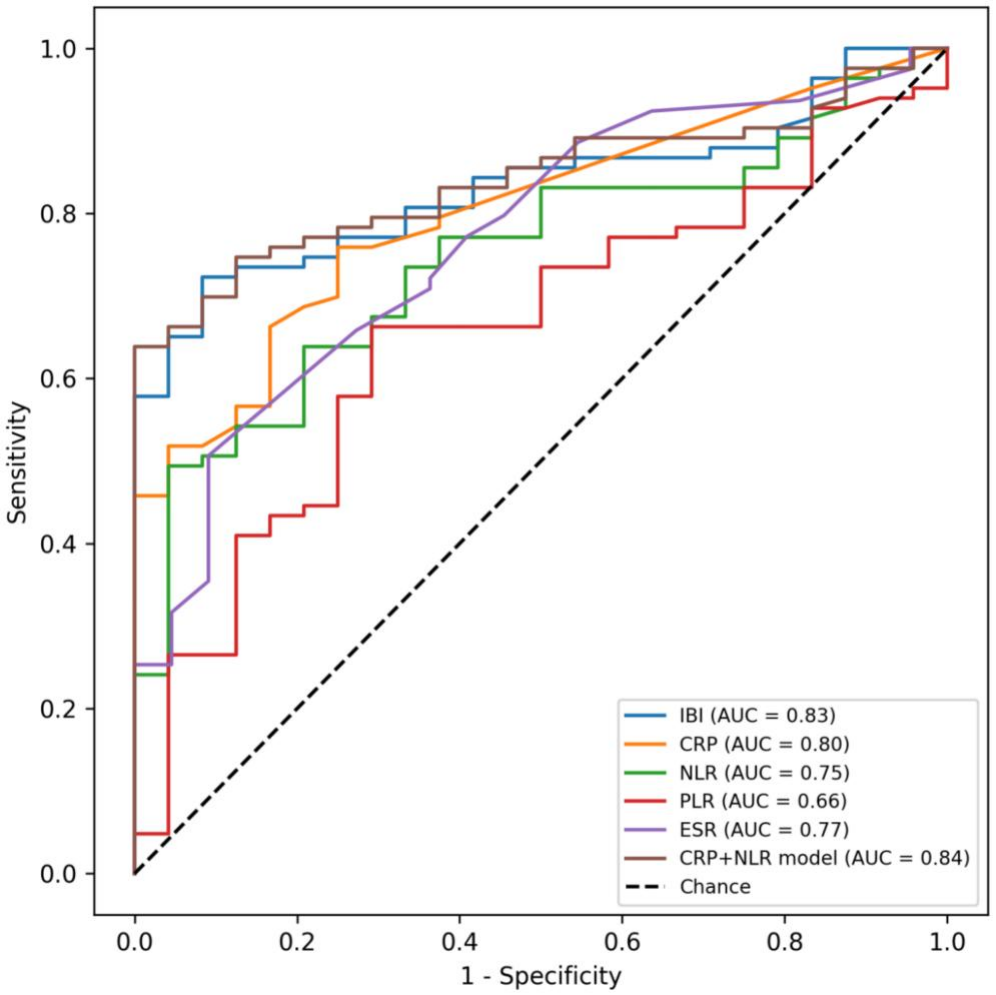
Receiver operating characteristic curves of IBI and systemic inflammatory markers for identifying moderate-to-severe endoscopic activity (MES ≥2). Receiver operating characteristic (ROC) curves for the inflammatory burden index (IBI, AUC = 0.83), a two-variable model combining CRP and NLR (AUC = 0.84), C-reactive protein (CRP, AUC = 0.80), erythrocyte sedimentation rate (ESR, AUC = 0.77; available in 101 patients), neutrophil-to-lymphocyte ratio (NLR, AUC = 0.75), and platelet-to-lymphocyte ratio (PLR, AUC = 0.66). Other markers were available for 107 patients.

We evaluated the discriminatory performance of IBI and conventional blood markers for identifying moderate-to-severe endoscopic activity (MES ≥2 vs MES 0-1) using receiver operating characteristic (ROC) analyses. IBI showed **reasonable** discrimination (AUC 0.83, 95% CI, 0.76-0.90). CRP alone also showed reasonable discrimination (AUC 0.80, 95% CI, 0.71-0.88), and same-sample DeLong testing indicated that the difference between IBI and CRP alone was not statistically significant (*P* = .227). A parsimonious two-variable logistic model combining CRP and NLR achieved comparable discrimination (AUC 0.84, 95% CI, 0.77-0.91) and was likewise not significantly different from IBI (DeLong *P* = .40). Other individual conventional markers showed lower point estimates of discrimination, including ESR (AUC 0.77, 95% CI, 0.65-0.87; measured in 101 patients), NLR (AUC 0.75, 95% CI, 0.65-0.84), and PLR (AUC 0.66, 95% CI, 0.54-0.77) (Table 4, Figure 3).

**Table 4:** Diagnostic performance of systemic inflammatory markers for identifying moderate-to-severe endoscopic activity (MES ≥2).

Marker	AUC (95% CI)	Optimal cutoff	Sensitivity, % (95% CI)	Specificity, % (95% CI)	LR+	LR−
**Inflammatory burden index (IBI)**	0.83 (0.76-0.90)	3.65	72.3 (61.8-80.8)	91.7 (74.2-97.7)	8.67	0.30
**CRP + NLR (two-variable model)**	0.84 (0.77-0.91)	NA	NA	NA	NA	NA
**C-reactive protein (CRP)**	0.80 (0.71-0.88)	0.88	75.9 (65.7-83.8)	75.0 (55.1-88.0)	3.04	0.32
**Erythrocyte sedimentation rate (ESR)**	0.77 (0.65-0.87)	18.00	50.6 (39.8-61.4)	90.9 (72.2-97.5)	5.57	0.54
**Neutrophil-to-lymphocyte ratio (NLR)**	0.75 (0.65-0.84)	3.34	49.4 (38.9-59.9)	95.8 (79.8-99.3)	11.86	0.53
**Platelet-to-lymphocyte ratio (PLR)**	0.66 (0.54-0.77)	148.06	66.3 (55.6-75.5)	70.8 (50.8-85.1)	2.27	0.48

Cutoff values were determined by maximizing Youden’s index (sensitivity + specificity − 1).

For the CRP+NLR model, sensitivity and specificity depend on the chosen probability threshold and are therefore not presented. The differences in AUC between IBI and CRP alone (*P* = .227) and between IBI and the CRP+NLR model (*P* = .40) were assessed using same-sample DeLong testing.

Abbreviations: AUC, area under the receiver operating characteristic curve; CI, confidence interval; CRP, C-reactive protein; ESR, erythrocyte sedimentation rate; IBI, inflammatory burden index; LR+, positive likelihood ratio; LR−, negative likelihood ratio; NLR, neutrophil-to-lymphocyte ratio; PLR, platelet-to-lymphocyte ratio.

Outcome: moderate-to-severe endoscopic activity (MES ≥2).

As shown in Table 4, the in-sample Youden-optimal cutoff on the original IBI scale was 3.65, yielding a sensitivity of 72.3% (95% CI, 61.8-80.8) and a specificity of 91.7% (95% CI, 74.2-97.7) for identifying MES ≥2. At this cutoff, the positive predictive value was 96.8% (95% CI, 89.0-99.1) and the negative predictive value was 48.9% (95% CI, 35.0-63.0). Given its relatively high specificity and modest sensitivity/negative predictive value in this cohort, the proposed cutoff is better viewed as a potential rule-in aid than a rule-out threshold. In this cohort, the Youden-derived IBI cutoff of 3.65 showed relatively high specificity. However, because this threshold was derived and evaluated within the same cohort, it should be interpreted as exploratory and cohort-specific. Overall, these results do not support clear superiority of IBI over CRP alone.

To address the potential influence of grouping MES 0 and MES 1 in the primary analysis, we additionally performed a sensitivity analysis using MES 3 as the endpoint. In this analysis, IBI remained significantly associated with severe endoscopic activity. The AUC of IBI for identifying MES 3 was 0.81 (95% CI, 0.72-0.89). In the age-, sex-, and BMI-adjusted model, log(IBI + 1) remained significantly associated with MES 3 (adjusted OR 2.50, 95% CI, 1.73-3.62; *P* < .001). These results are also shown in Table S3.

### Sensitivity analysis according to the observed blood-draw-to-endoscopy interval

**Figure 4:**
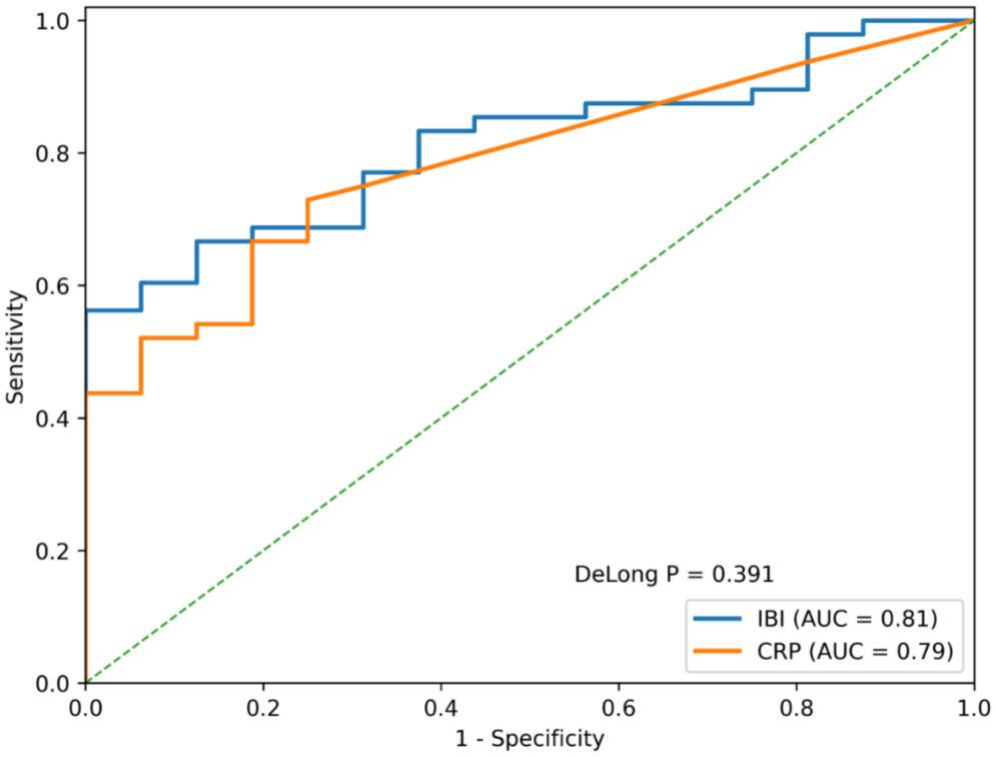
Receiver operating characteristic curves for IBI and CRP in patients who underwent index endoscopy within 48 hours after blood sampling. In the subgroup of hospitalized patients with ulcerative colitis who underwent index endoscopy within 48 hours after blood sampling (*n* = 64), IBI showed an AUC of 0.81, compared with 0.79 for CRP alone; the difference was not statistically significant on same-sample DeLong testing (*P* = .391).

As a secondary timing analysis, the observed blood-draw-to-endoscopy interval was available as categorical windows rather than exact timestamps, with 19 patients undergoing index endoscopy within 24 hours after blood sampling, 45 between >24 and 48 hours, and 43 between >48 and 72 hours. In a sensitivity analysis restricted to patients who underwent index endoscopy within 48 hours after blood sampling (*n* = 64), the association between log(IBI + 1) and moderate-to-severe endoscopic activity (MES ≥2) remained significant in both the unadjusted model (OR 3.97, 95% CI, 1.50-10.48; *P* = .0055) and the model adjusted for age, sex, and BMI (adjusted OR 4.51, 95% CI, 1.59-12.79; *P* = .0047). Discriminatory performance was also broadly preserved in this restricted subset, with an AUC of 0.81 for IBI compared with 0.79 for CRP alone (same-sample DeLong *P* = .391). Using the cohort-derived cutoff of 3.65 from the full cohort, IBI yielded a sensitivity of 66.7% and specificity of 87.5% in the ≤48 hours subset (Figure 4; Table S4).

### Internal validation and calibration results

Because standalone IBI was the primary index of interest, we additionally performed bootstrap resampling (5000 resamples) to assess its discrimination and cutoff stability directly. The bootstrap median AUC was 0.83 (95% bootstrap interval 0.75-0.90). For the overall in-sample cutoff of 3.65 on the original IBI scale, the bootstrap median sensitivity was 72.5% (95% bootstrap interval 62.7%-81.8%), and the bootstrap median specificity was 92.0% (95% bootstrap interval 78.9%-100.0%). The bootstrap median Youden-optimal cutoff was 3.655 (IQR 3.655-5.221), suggesting some within-cohort stability of the derived threshold, although variability was present. These results are presented in Table S5.

As a supplementary model-level analysis, the multivariable model including log(IBI + 1), age, sex, and BMI demonstrated an apparent AUC of 0.85. In bootstrap internal validation (500 resamples), the estimated optimism in AUC was 0.026, yielding an optimism-corrected AUC of 0.82. Calibration after optimism correction was also acceptable: the calibration intercept was 0.07, indicating minimal systematic miscalibration, and the calibration slope was 0.83, suggesting some overfitting such that predicted risks were slightly more extreme than the observed event rates. Additional internal validation and calibration metrics for the multivariable model are provided in Table S6.

### Decision-curve analysis

Decision-curve analysis was performed to evaluate net benefit for standalone IBI, CRP alone, and the multivariable model in identifying moderate-to-severe endoscopic activity (MES ≥2). Across threshold probabilities from 0 to 0.80, standalone IBI and CRP alone showed broadly comparable net benefit, whereas the multivariable model showed similar or modestly greater net benefit over part of the threshold range. These results are shown in Figure S1.

## Discussion

In this single-center retrospective study of hospitalized patients with UC, the IBI was associated with endoscopic activity in the inpatient setting. IBI showed the highest Spearman correlation coefficient with the MES among the evaluated blood indices and remained independently associated with moderate-to-severe endoscopic activity (MES ≥2) after adjustment for age, sex, and BMI. In ROC analyses, IBI showed reasonable discrimination for MES ≥2, and the cohort-derived cutoff showed high specificity with moderate sensitivity in this dataset. However, the incremental gain over CRP alone was modest and not statistically significant on same-sample DeLong testing, and these findings should therefore not be interpreted as demonstrating clear superiority over CRP alone.

Our findings are consistent with prior work showing that conventional blood inflammatory markers correlate only moderately with mucosal activity in UC.^5^^,^^14^^,^^24^ Leukocyte-derived ratios such as NLR and PLR are inexpensive and readily available, but their diagnostic performance has been variable across cohorts and is often insufficient when used as standalone indicators.^14^^,^^25^^,^^26^ IBI summarizes CRP- and NLR-related inflammatory information in a single composite measure. A parsimonious CRP+NLR model achieved discrimination comparable to IBI, suggesting that IBI largely reflects the joint information conveyed by these two routinely obtained measures. In our cohort, the Youden-derived cutoff for CRP was 0.88 mg/L, which was below the local laboratory upper limit of normal (10 mg/L), suggesting that endoscopically relevant inflammatory activity may be present even when CRP remains within the conventional reference range. Because IBI combines CRP with NLR on a single composite scale, it may pragmatically summarize routinely available inflammatory information; however, this should not be interpreted as evidence of superior clinical utility over CRP alone. Because IBI incorporates CRP as one of its components, its performance may be attenuated in patients with active UC who mount a limited CRP response despite endoscopic inflammation. Accordingly, a low IBI value should not be interpreted as excluding moderate-to-severe endoscopic activity. In this cohort, the IBI cutoff showed relatively high specificity; however, because it was derived and evaluated within the same cohort, it should be interpreted as exploratory and cohort-specific rather than as an established action threshold.

Several limitations warrant consideration. First, FC—a benchmark noninvasive biomarker for UC activity—was not routinely measured in this inpatient cohort, precluding a direct comparison.^8^^,^^27^^,^^28^^,^^29^ FC is well validated and often outperforms conventional blood markers in correlating with endoscopic activity and mucosal healing.^4^^,^^28^^,^^30^ However, its use in hospitalized patients is often limited by practical challenges such as delayed stool collection and laboratory turnaround times.^29^^,^^31^ Accordingly, our findings should be interpreted in the context of an inpatient setting in which FC is not routinely available, rather than as establishing a substitute for FC in outpatient monitoring. Future studies directly comparing IBI and FC in hospitalized cohorts will be important to clarify how these measures compare and whether they provide complementary information.

Second, this was a retrospective, single-center study with a high prevalence of MES ≥2, which reflects the inpatient flare population but may limit generalizability to settings with lower disease acuity. In addition, because the cohort was restricted to hospitalized UC patients who underwent endoscopic evaluation during the index admission, it represents an endoscopically selected subgroup rather than the broader hospitalized UC population. Such patients may have had greater clinical concern for active disease, higher inflammatory burden, or a stronger indication for endoscopic assessment, potentially contributing to selection bias and spectrum bias and limiting extrapolation of the proposed cutoff to less selected inpatient populations.

Furthermore, predictive values are prevalence-dependent and may differ in lower-acuity populations. The proposed IBI threshold was derived within this cohort and may require recalibration in populations with different disease spectra, treatment patterns, or baseline inflammatory profiles. Although endoscopic scoring was blinded to laboratory results and intra-rater reliability was high, MES was assigned retrospectively based on endoscopy reports and available images, and histologic activity was not assessed. In addition, endoscopic assessment in routine inpatient practice included both full colonoscopy and flexible sigmoidoscopy, which may have introduced some heterogeneity in examined extent despite the use of standardized MES scoring rules. Finally, although age, sex, and BMI were adjusted for in multivariable analyses, residual confounding from unmeasured clinical factors cannot be excluded. Major comorbid conditions likely to substantially affect systemic inflammatory markers were excluded a priori, but unmeasured sources of confounding may still have remained. Although blood samples were collected within 24 hours of hospital admission and index endoscopy was performed within 72 hours of hospital admission, exact continuous timing data were not consistently available across the full cohort. Both assessments were anchored to the same hospitalization-related inflammatory episode, and the observed blood-draw-to-endoscopy interval was analyzed separately as a secondary timing variable to assess robustness to narrower sampling windows. Baseline/admission treatment background was summarized to characterize the clinical context. However, because the precise sequencing of treatment changes between blood draw and endoscopy could not be reliably determined from the retrospective source records, temporal mismatch and treatment-related confounding cannot be excluded.

Recent efforts have continued to develop pragmatic approaches for assessing UC activity,^32^ and data-driven models using routinely collected laboratory data have also been explored for noninvasive prediction of endoscopic activity.^33^^,^^34^ In this context, the present study examined a simple composite index derived from routinely available blood markers in a hospitalized UC cohort. The bootstrap validation and calibration assessment in this study pertained to the multivariable model including log(IBI + 1), age, sex, and BMI, and should therefore be interpreted as supplementary model-level analyses rather than direct validation of the standalone IBI cutoff itself. In addition, decision-curve analyses for standalone IBI and CRP alone showed broadly comparable net benefit. Any numerical differences between standalone IBI and CRP alone should be interpreted descriptively. To further address this distinction, we additionally performed bootstrap-based internal assessment of the standalone IBI itself, which showed generally consistent discrimination and a cohort-specific cutoff distribution centered around 3.65. Prospective multicenter validation remains essential.

In conclusion, IBI is a simple and readily available composite index derived from routine blood tests that is associated with endoscopic activity in hospitalized UC. In this endoscopically selected inpatient cohort, its discriminatory performance was comparable to that of CRP alone, and the cohort-derived cutoff should be regarded as exploratory and interpreted cautiously given the retrospective design, incomplete treatment-timing data, and limited granularity of blood-to-endoscopy timing data. Prospective multicenter studies—ideally including FC, broader severity distributions, standardized endoscopic assessment, and systematically recorded treatment timing—are warranted before broader clinical application.

## Author contributions

Conceptualization: Xiaoyun Wang; Methodology: Zihan Xu; Formal analysis: Zihan Xu and Shiqing Yuan; Investigation: Zhen Hu, Jiarun Qian, and Lihong Gu; Resources: Zhen Hu and Lihong Gu; Data curation: Zihan Xu, Shiqing Yuan, and Jiarun Qian; Writing—original draft preparation: Zihan Xu and Shiqing Yuan; Writing—review and editing: Zhen Hu, Lihong Gu, and Xiaoyun Wang; Visualization: Zihan Xu and Jiarun Qian; Supervision: Xiaoyun Wang; Project administration: Xiaoyun Wang. All authors have read and agreed to the published version of the manuscript.

## Supplementary Material

otag073_Supplementary_Data

## Data Availability

The data presented in this study are available on request from the corresponding author. The data are not publicly available due to privacy and ethical restrictions.
