# Supplementary material for: Inflammatory burden index (CRP×NLR) is associated with moderate-to-severe endoscopic activity in hospitalized patients with ulcerative colitis: a single-center retrospective study
Source: Crohns Colitis 360. 2026 Aug 8;8(3):otag073. doi: 10.1093/crocol/otag073 (PMC13459971; doi:10.1093/crocol/otag073)
Supplement: Supplementary file 1 [file otag073_supplementary_data.docx]

Supplementary Materials：

Supplementary Table S1

Full distribution of Mayo Endoscopic Subscores

| **MES category** | **n (%)** |
| --- | --- |
| MES 0 | 7 (6.5%) |
| MES 1 | 17 (15.9%) |
| MES 2 | 35 (32.7%) |
| MES 3 | 48 (44.9%) |

Supplementary Table S2.

Distribution of blood-draw-to-endoscopy intervals in the analytic cohort

| Blood-draw-to-endoscopy interval | n (%) |
| --- | --- |
| Within 24 h | 19 (17.8%) |
| >24 to 48 h | 45 (42.1%) |
| >48 to 72 h | 43 (40.2%) |
| Total | 107 (100.0%) |

Note: All included patients had blood samples collected within 24 h of hospital admission. The blood-draw-to-endoscopy interval was available in the structured dataset and was categorized as within 24 h, >24 to 48 h, and >48 to 72 h after blood sampling.

Supplementary Table S3. Sensitivity analysis using MES 3 as the endpoint

| **Analysis** | **Endpoint** | **n** | **Measure** | **Value (95% CI)** | **P value** |
| --- | --- | --- | --- | --- | --- |
| ROC analysis | MES 3 | 107 | IBI AUC | 0.81 (0.72–0.89) | — |
| Adjusted logistic regression | MES 3 | 106 | log(IBI + 1), adjusted OR | 2.50 (1.73–3.62) | <0.001 |

Adjusted logistic regression was performed in complete cases with available age, sex, BMI, and IBI (n = 106).

Supplementary Table S4. Sensitivity analysis restricted to patients who underwent index endoscopy within 48 h after blood sampling

| **Analysis** | **Estimate** | **95% CI** | **P value** |
| --- | --- | --- | --- |
| Association with moderate-to-severe endoscopic activity (MES ≥2) |  |  |  |
| log(IBI + 1), univariable OR | 3.97 | 1.50–10.48 | 0.0055 |
| log(IBI + 1), adjusted OR* | 4.51 | 1.59–12.79 | 0.0047 |
| Discriminatory performance for MES ≥2 |  |  |  |
| IBI, AUC | 0.81 | — | — |
| CRP, AUC | 0.79 | — | — |
| IBI vs CRP, same-sample DeLong test | — | — | 0.391 |
| Performance of the overall in-sample IBI cutoff in the ≤48 h subset |  |  |  |
| IBI cutoff (original scale) | 3.65 | — | — |
| Sensitivity, % | 66.7 | — | — |
| Specificity, % | 87.5 | — | — |

Abbreviations: AUC, area under the receiver operating characteristic curve; CI, confidence interval; CRP, C-reactive protein; IBI, inflammatory burden index; MES, Mayo Endoscopic Subscore; OR, odds ratio.

*Adjusted for age, sex, and body mass index.

The ≤48 h subgroup included 64 patients who underwent index endoscopy within 48 h after blood sampling. The cutoff of 3.65 was the overall in-sample Youden-optimal cutoff on the original IBI scale and was applied to the ≤48 h subset for sensitivity analysis.

Supplementary Table S5. Bootstrap-based internal assessment of standalone IBI discrimination and cutoff stability

| **Parameter** | **Value** |
| --- | --- |
| Bootstrap resamples | 5,000 |
| Bootstrap median AUC | 0.834 |
| 95% bootstrap interval for AUC | 0.753–0.902 |
| In-sample Youden-optimal cutoff (full cohort) | 3.65 |
| Bootstrap median sensitivity at cutoff 3.65 | 72.5% |
| 95% bootstrap interval for sensitivity | 62.7%–81.8% |
| Bootstrap median specificity at cutoff 3.65 | 92.0% |
| 95% bootstrap interval for specificity | 78.9%–100.0% |
| Bootstrap median Youden-optimal cutoff | 3.655 |
| IQR of bootstrap optimal cutoff | 3.655–5.221 |

Note: Bootstrap resampling was performed 5,000 times using the full analytic cohort. Standalone IBI was assessed for discrimination against MES ≥2. Cutoff stability was evaluated for the cohort-derived threshold of 3.65 and by the distribution of bootstrap Youden-optimal cutoffs.

Supplementary Table S6. Internal validation and calibration metrics for the multivariable model predicting moderate-to-severe endoscopic activity

| Metric | Estimate |
| --- | --- |
| Apparent AUC | 0.85 |
| Estimated optimism | 0.026 |
| Optimism-corrected AUC | 0.82 |
| Calibration intercept | 0.07 |
| Calibration slope | 0.83 |

**Abbreviations:** AUC, area under the receiver operating characteristic curve; BMI, body mass index; IBI, inflammatory burden index.

**Note:** The multivariable model included log(IBI + 1), age, sex, and BMI and was fitted in complete cases (n = 106). Internal validation was performed using 500 bootstrap resamples.

Supplementary Figure S1.

A

B

Supplementary Figure S1. Decision-curve analysis. (A) Decision-curve analysis comparing standalone IBI and CRP alone for identifying moderate-to-severe endoscopic activity (MES ≥2). Standalone IBI showed net benefit broadly comparable to CRP alone across the examined threshold probabilities. (B) Decision-curve analysis for the multivariable model including log(IBI + 1), age, sex, and BMI for identifying moderate-to-severe endoscopic activity (MES ≥2). The multivariable model showed similar or modestly greater net benefit over part of the examined threshold range.
